# Biomedical word sense disambiguation with bidirectional long short-term memory and attention-based neural networks

**DOI:** 10.1186/s12859-019-3079-8

**Published:** 2019-12-02

**Authors:** Canlin Zhang, Daniel Biś, Xiuwen Liu, Zhe He

**Affiliations:** 1 Department of Mathematics, Florida State University, Tallahassee, FL, US; 2 Department of Computer Science, Florida State University, Tallahassee, FL, US; 3 School of Information, Florida State University, Tallahassee, FL, US

**Keywords:** Word sense disambiguation, LSTM, Self-attention, Biomedical

## Abstract

**Background:**

In recent years, deep learning methods have been applied to many natural language processing tasks to achieve state-of-the-art performance. However, in the biomedical domain, they have not out-performed supervised word sense disambiguation (WSD) methods based on support vector machines or random forests, possibly due to inherent similarities of medical word senses.

**Results:**

In this paper, we propose two deep-learning-based models for supervised WSD: a model based on bi-directional long short-term memory (BiLSTM) network, and an attention model based on self-attention architecture. Our result shows that the BiLSTM neural network model with a suitable upper layer structure performs even better than the existing state-of-the-art models on the MSH WSD dataset, while our attention model was 3 or 4 times faster than our BiLSTM model with good accuracy. In addition, we trained “universal” models in order to disambiguate all ambiguous words together. That is, we concatenate the embedding of the target ambiguous word to the max-pooled vector in the universal models, acting as a “hint”. The result shows that our universal BiLSTM neural network model yielded about 90 percent accuracy.

**Conclusion:**

Deep contextual models based on sequential information processing methods are able to capture the relative contextual information from pre-trained input word embeddings, in order to provide state-of-the-art results for supervised biomedical WSD tasks.

## Background

In the health and biomedical domain, valuable information can be mined from a huge amount of unstructured data, such as scientific literature, clinical narratives in the electronic health records, and health-related postings on social media [1]. Similar to natural language processing (NLP) in the general domain, knowledge discovery and information extraction require specialized tasks such as syntactic parsing, named entity recognition (NER), and relation extraction. In NER, an important step is to decide the correct sense of an ambiguous word or phrase based on its context. Otherwise, the accuracy of downstream NLP applications such as sentiment analysis and text classification will suffer.

In the biomedical NLP area, well-curated medical terminologies and lexicons lay a solid foundation. The Unified Medical Language System (UMLS), which consists of over 200 biomedical terminologies and ontologie, has more than ten million terms and three million concepts. The terms with the same meaning are mapped to the same concepts. For example, *myocardial infarction* and *heart attack* are mapped to the same concept, which is assigned a concept unique identifier (CUI). Biomedical NER is usually realized by correctly recognizing and mapping an entity mentioned in the sentence to a concept in the UMLS. For instance, the term *nursing* has two concepts in the UMLS: *Discipline of Nursing* and *Breast Feeding*. In the sentence *“Breastfeeding is in general safe but needs appropriate observation of the nursing infant.”nursing* refers to *Breast Feeding*, whereas in another expression *“Strategic research, technological innovation and nursing”*, *nursing* refers to *Discipline of Nursing*.

Biomedical texts often contain a series of lexical ambiguities, such as abbreviations and polysemous terms. For instance, the acronym *CRF* may refer to *chronic renal failure*, or *corticotropin-releasing factor*. Some terms have different but very similar meanings. For instance, *malaria* may refer to the disease malaria, or the malaria vaccine. When extracting information from biomedical texts, selecting the correct meaning (“sense”) for an ambiguous term based on its context is called word sense disambiguation (WSD) [2].

Biomedical WSD has been a long-standing challenge for more than 20 years. Many biomedical WSD methods have been developed. As far as 2004, Liu et al. evaluated supervised methods including decision lists [3] and Naive Bayes. In 2006, Xu et al. [4] improved the supervised approaches and indicated that the error rate of supervised approaches is proportional to the similarity of senses. Also, it is very expensive to generate a labeled corpus. Researchers tried to reduce the labeling costs by several different approaches. Wang et al. [5] proposed an interactive learning method to reduce labeling expense while outperforming the active learning approach. Semi-supervised learning is another type of approaches. In Liu et al. [6], labeled data is first generated automatically from UMLS and MEDLINE databases, then used to train supervised algorithms. Similarly, Yu et al. used MEDLINE abstracts to create labeled data for their supervised training algorithms [7]. Besides these efforts, Xu et al. leveraged knowledge from dictated dispatch discharge in the clustering analysis and estimated sense frequency for WSD [8]. In addition, Duque et al. completed the same task by incorporating external knowledge resource. They leveraged co-occurrence information in a graph-based unsupervised WSD method [9]. Yepes et al. [10] conducted a study comparing four different knowledge based methods. The best results were obtained via a WSD method using the semantic types assigned to the concepts in the UMLS Metathesaurus. The context of the ambiguous word and semantic types of the candidate concepts are mapped to journal descriptors. The journal descriptors are compared to choose among the candidate concepts. Sabbir et al. [11] used a concept mapping tool MetaMap to label a corpus of PubMed abstracts with UMLS CUIs. They used the Word2Vec algorithm [12] to generate concept embeddings. They used cosine similarity and K-NN algorithm to disambiguate the words in the MSH WSD dataset, reporting state-of-the-art results achieved by an unsupervised system. However, Sabbir et al. pointed out that their approach may be considered weakly supervised because of the use of MetaMap. Rais et al. [13] introduced No Distance Sense Relate, a modification of the Sense Relate Algorithm. No Distance Sense Relate ignores the distance of the context word from the word being disambiguated, therefore all the terms in the context have an equal weight. No Distance Sense Relate method, evaluated on the MSH WSD dataset, consistently yielded a higher accuracy with a window size of 3. However, with a window size of 2, Sense Relate method yielded a higher disambiguation accuracy than No Distance Sense Relate. It was concluded that depending on the window size, the distance between the target word and the terms in the context can influence the accuracy of the model differently. Recently, with the promise of deep neural networks in NLP tasks, specialized neural network architectures have been developed for WSD. Typical approaches include the recurrent convolutional neural networks evaluated by Festag and Spreckelsen [14], and the LSTM network proposed by Yepes [15]. These approaches indicate that a large amount of high-quality, annotated data is required to achieve satisfactory performance in WSD.

To infer the correct sense of an ambiguous word, existing WSD methods often leverage the information of the context. However, merely focusing on the co-occurrence of ambiguous words may not be sufficient to determine their correct senses. Consider the word *contract* in this example: *“Since one of the workers contracted leukemia, the company hired a well-known law firm to protect itself in case of the potential lawsuit,”*. The presence of “workers”, “company”, “firm” and “law” may overweight the word “leukemia”, indicating that *to contract* in this context means *making a legal agreement with someone*, rather than its correct sense *catching or becoming ill with a disease*. We thus believe that it is necessary to use a sequential information processing system to resolve this issue and capture semantic relations in the context. Moreover, the local context of the target ambiguous word may not provide enough information for WSD. Say, in order to decide the correct meaning (sense) of the word *nursing* in the sentence *“The absolute need to articulate the gender issue in nutrition, nursing and medical academic curricula is stated.”*, a large context is required even for humans. Typically, a suitable context should be part of the paragraph. Since most paragraphs in our dataset are usually within 200 words, we will use the entire paragraph to infer the meaning of each ambiguous term.

In this work, we apply deep contextual representations [16] to establish two supervised WSD systems: (1) a WSD system based on both a multi-layer bidirectional Long Short-Term Memory (BiLSTM) neural network model, and (2) a WSD system based on the self-attention model. Note that this work is based on our previous work [17]: We apply the same BiLSTM neural network model from [17], but we further build an attention model with the self-attention architecture introduced in [18]. Also, we further expand our previous research on universal WSD systems in this work. The contributions of this work are three-fold:

First, we build a deep contextual representation of a target ambiguous word, using the output from two layers of the BiLSTM network or the attention architecture. In contrast to [16], in which weighted summation of the lower layers is applied, we perform a max-pooling operation to extract related features from the context. Our models take pre-trained word embeddings as inputs. However, note that our BiLSTM network and attention model are not pre-trained on any other general NLP datasets, but trained end-to-end on the WSD dataset.

Second, our contextual representation is not only “deep” but also “wide”: We use the outputs at multiple time steps, rather than only using the output at time-step *t*, where **x**_*t*_ is the target word embedding. Then, we perform the max-pooling operation along the time-step. As our results show, larger and wider contexts lead to significant improvements in prediction accuracy.

Third, we make efforts to develop universal models for biomedical WSD. All the existing methods (e.g., [14, 15]) build a model for each ambiguous word separately. In contrast, we design universal WSD models by concatenating the embedding of the target ambiguous word with the max-pooled output. Then we train both the BiLSTM neural network model and the self-attention model on all the words in the dataset. Experimental results show that this concatenation design significantly increases the prediction accuracy of our universal BiLSTM neural network model. Furthermore, the prediction accuracy on some words are higher when using the universal network than the word-specific ones.

The rest of this paper is organized as follows: We provide a detailed explanation on both of our models in the “Methods” section, in which all the four structures and the hint layer are discussed in detail. In the “Results” section, the results and operating details of our experiments are provided. Then, we provide a comprehensive analysis on our models in the “Discussion” section, including the advantages and potential weaknesses of the BiLSTM neural network model against the self-attention model. Finally, we conclude this work with a summary and our future research plans in the last section.

## Methods

In this section, we shall introduce our methods in detail. We provide two approaches: a WSD method based on a BiLSTM neural network, and a WSD method based on the self-attention model introduced in [18].

Note that both of our BiLSTM neural network model and our attention model share the same upper layer structure: We designed four different transformation structures to operate on the outputs of the two BiLSTM layers or the two attention layers (both our BiLSTM model and our attention model are always a stack of two identical layers). Then, a max-pooling layer shall operate on the chosen transformation structure to generate a dense embedding. Finally, an optional concatenation between the target ambiguous word embedding and the dense output embedding may be implemented before the softmax result.

The novelty of our work lies in the four transformation structures and the optional concatenation in the upper layer. We will introduce this architecture together with the BiLSTM model in the first subsection. Then in the second subsection, we will provide a detailed introduction to the attention architecture.

### WSD Method based on the BiLSTM Network

In this subsection, we shall first introduce the structure of an LSTM cell. Then, our adjustments to the LSTM output and the concatenation of the target word embedding to the max-pooled vector in the upper layer of our neural network model are introduced in detail.

#### Long Short-Term Memory Networks

**Fig. 1:**
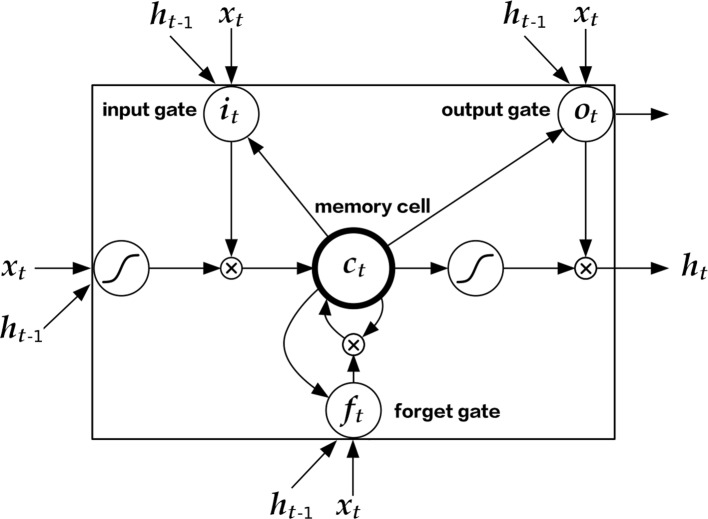
The structure of an LSTM cell. This figure comes from [26], with minor adjustments applied

**Long Short-Term Memory** (LSTM) is a gated Recurrent Neural Network (RNN) introduced by Hochereiter and Schimdhuber in 1997 [19] and refined by Gers in 1999 [20]. The structure of an LSTM cell is shown in Fig. 1. Mathematically, the operation within an LSTM cell can be described as:

1 $$ i_{t} = \sigma(W_{{xi}}x_{t} + W_{{hi}}h_{t-1} +b_{i})  $$

2 $$ f_{t} = \sigma(W_{{xf}}x_{t} + W_{{hf}}h_{t-1} +b_{f})  $$

3 $$ c_{t} =f_{t}c_{t-1}+i_{t}\tanh(W_{{xc}}x_{t} + W_{{hc}}h_{t-1} + b_{c})  $$

4 $$ o_{t} = \sigma(W_{{xo}}x_{t} + W_{{ho}}h_{t-1}+ b_{o})  $$

5 $$ h_{t} = o_{t}\tanh(c_{t})  $$

Here, *σ* represents the sigmoid function: *σ*(*x*)=1/(1+ exp(−*x*)). And as we mentioned above, there are three “gates” operating in an LSTM cell: The forget gate is denoted as **f**_*t*_; the input gate as **i**_*t*_; and output gate as **o**_*t*_. Within an LSTM cell, the three gates shall operate with the trainable matrices *W* to keep “valuable” information from previous time steps and eliminate “invaluable” parts according to the provided label. This operation is a recurrent process, which has been introduced in detail in our previous paper [17]. For brevity, we will not repeat the details here.

Thanks to the ability to capture long-term semantic dependencies and the superior performance on long sequences, the LSTM is commonly used in many NLP tasks. In this work, we will use a specific type of LSTM, the **Bidirectional LSTM** (BiLSTM) [21]. In case of BiLSTM, the input sequence will be processed in both forward and backward directions, with independent parameters in each direction. The outputs at each time-step from both directions are concatenated and become the input of the BiLSTM in the next layer, in case of multiple layers. As such, the complete information about the whole input sequence will be captured by the neural network node at any time-step. In order to take advantage of this feature, we use BiLSTM networks to better capture the semantic relations on both sides of the target word.

#### Structure of the upper layer

We use the BiLSTM neural network model as the example to show the structure of the upper layer. As we mentioned above, the BiLSTM neural network model and the attention model share the same upper layer structure. In order to build the complete architecture of our attention model, one only needs to replace each BiLSTM layer with an attention layer, keeping all the other structures unchanged.

For the training of the BiLSTM neural network, we use 25 words before and after the target ambiguous word as the input in order to reduce training time. As shown in the Results subsection, the overall performance is improved when the network is trained with full paragraphs. Suppose the first layer output is **Y**=(**y**_1_,⋯,**y**_*T*_), and the second layer output is **Z**=(**z**_1_,⋯,**z**_*T*_), with **y**_*i*_ and **z**_*i*_ to be vectors with the same dimension $\mathcal {D}$, i.e., $\mathbf {y}_{i},\mathbf {z}_{i}\in \mathbb {R}^{\mathcal {D}}$. And after applying different layer settings, we decide that a two-layer BiLSTM with dropout [22] provides the best performance.

We design in total four optional structures to perform on top of the BiLSTM to adjust its output. We use **H** to represent the output of each structure. Then, these four structures can be described as the followings:

(i) We directly use the output from the BiLSTM. That is, **H**=**Z**.
(ii) We perform weighted summation between **Y** and **Z**. That is, **H**=*λ***Y**+(1−*λ*)**Z**, where *λ*∈[0,1] is a variable.
(iii) We concatenate **Y** and **Z** along time steps. That is, since both **Y** and **Z** are $T\times \mathcal {D}$ tensors, **H** will be a $2T\times \mathcal {D}$ tensor.
(iv) We concatenate **Y** and **Z** along each vector **y** and **z**. That is, **H** will be a $T\times 2\mathcal {D}$ tensor.

After a specific upper layer structure is chosen, we perform a max-pooling operation along time-steps on **H** to get $\mathbf {h} \in \mathbb {R}^{\mathcal {D}}$ (or $\mathbb {R}^{2\mathcal {D}}$ in case of structure (iv)). That is, we pick the maximum value along the time-steps within each dimension $d \in \mathcal {D} \ (\text {or} \ 2\mathcal {D})$. Based on these settings, we hope that the context vector **h** can capture the context information that is sufficient for disambiguation.

**Fig. 2:**
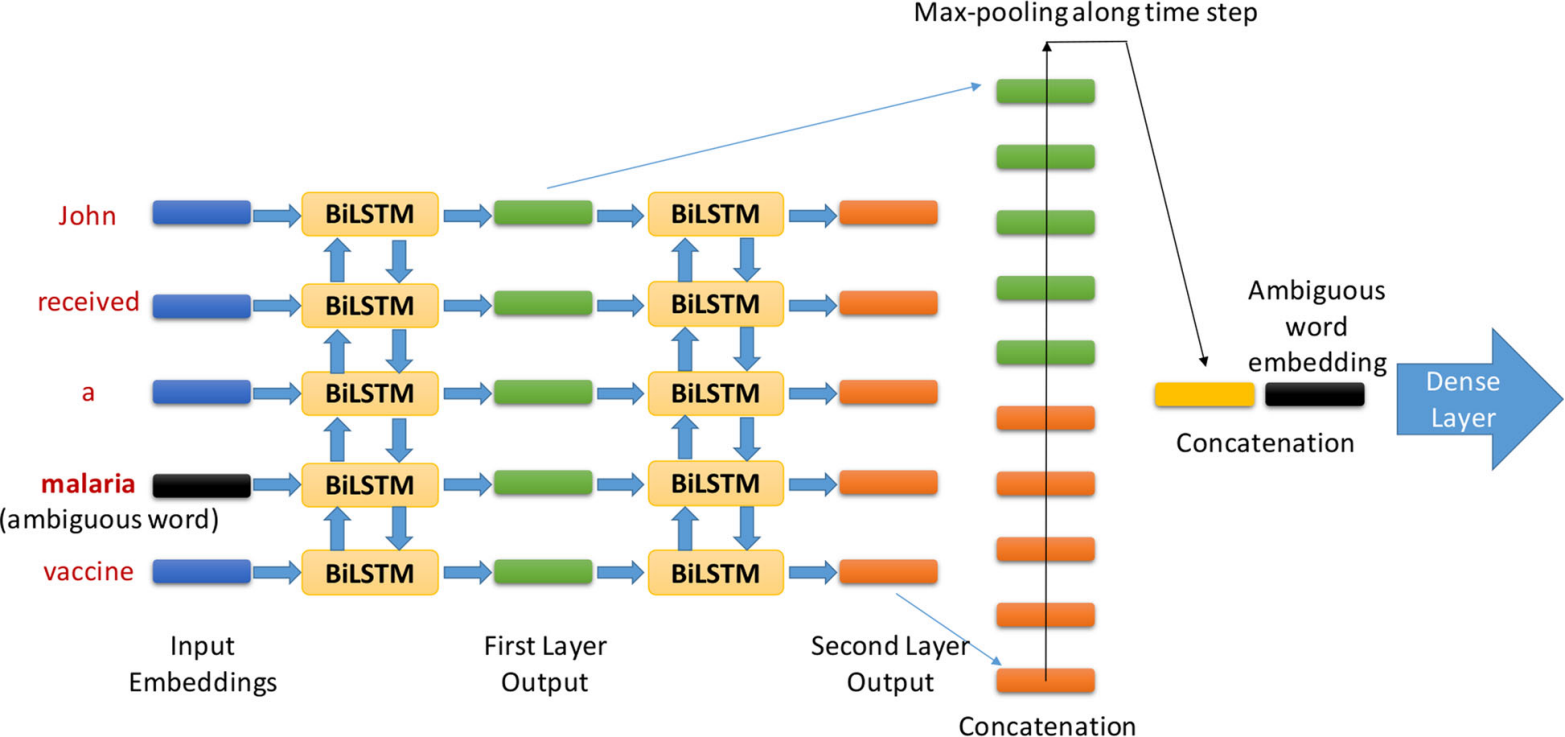
Contextual dependent neural network with the concatenation of LSTM outputs along time-step, as well as the optional layer $\mathcal {C}$. This graph comes from our previous paper [17]

In addition, an optional step $\mathcal {C}$ is provided: The target word embedding **x**_*k*_ and the context vector **h** will be concatenated to form the **context** **−** **word** **embedding** **ξ**=[**h**,**x**_*k*_]. Finally, the vector **ξ** (or **h** if the optional layer is not applied) passes through two dense layers with 256 and 64 hidden units respectively, before the softmax output. We will provide experimental results from neural network models with or without the optional layer $\mathcal {C}$. The results confirm our assumption that the optional layer $\mathcal {C}$ is more beneficial in case of the universal model than the word-specific model, which is discussed in the “Discussion” section. The complete network structure is shown in Fig. 2. For clarity, we only specify the version (iii) in it.

#### Training

In each training step, we give a paragraph (*w*_1_,⋯,*w*_*T*_) with the target ambiguous word *w*_*t*_ marked out. There is only one target ambiguous word in each paragraph. For an ambiguous word *w*, its possible senses are labeled as *M*_1_,*M*_2_, etc. The correct sense *M*_*i*_ of *w* of the ambiguous word is given at the end of the paragraph. The label set {*M*_*i*_} is shared by all ambiguous words, which means the label itself does not specify a UMLS concept, and contains no semantic information.

Then, the corresponding pre-trained word embedding (**x**_1_,⋯,**x**_*T*_) is obtained from the embedding matrix accordingly. We either use the full paragraph as the input, or use a fixed-length input with 25 words before and after **x**_*t*_, i.e., (**x**_*t*−25_,⋯,**x**_*t*−1_,**x**_*t*_, **x**_*t*+1_,⋯,**x**_*t*+25_). For simplicity, we mostly use *X*=(**x**_1_,⋯,**x**_*T*_) to represent the sequence in this paper without specifying what kind of input method we use. We shall specify it when necessary.

#### Implementation details

We applied exponential decayed learning rate: Starting from 0.05, the learning rate decays every 2500 steps with a base equals to 0.96. We used Adagrad Optimizer due to its suitability for training on sparse data and its ability to perform more informed gradient-based learning [23]. In addition, we used an early stopping technique in order to make learning process more time-efficient. Since the target ambiguous words in the MSH WSD dataset do not have the same number of training items, we decided to save a checkpoint when the lowest validation loss is noted. We restored the checkpoint and stopped the training if the prediction accuracy did not decrease after 5 epochs on the validation set. This method applies dynamic training epochs and hence makes the training more flexible for different input files. All our models were implemented in TensorFlow [24].

### WSD Methods based on the Attention Model

Our attention model has the same upper layers as our BiLSTM neural network model. That is, as we mentioned, we only use the attention layer to replace the BiLSTM network layer when switching from the BiLSTM neural network model to the attention model. The structures (i) through (iv), the max-pooling layer and the optional layer $\mathcal {C}$ are all the same. We shall note that the attention architecture in this work is mainly based on the self-attention encoder and decoder in [18]. To be specific, we only apply the encoder architecture from [18], since our WSD task is not complicated enough to apply the decoder. One can find an overall discussion on the self-attention encoder and decoder models in [18].

We shall use five parts in this subsection to present the attention architecture used in this paper: Part one serves as a general introduction, indicating that our attention architecture is actually a stack of several identical attention layers (or identical encoder layers). Part two introduces the scaled dot-product attention, the core attention mechanism in an encoder layer. Part three indicates that in each encoder layer, a number of scaled dot-product attentions shall operate in parallel to provide multiple outputs, which will then be concatenated and projected into one final attention output. This process is called multi-head self-attention (or multi-head attention in short). Part four shows the structure of the feed-forward network, which is located on top of the multi-head attention within an encoder layer. Finally, part five indicates how the order of sequence of the input words is encoded into the input embeddings.

#### General attention architecture: A stack of identical layers

**Fig. 3:**
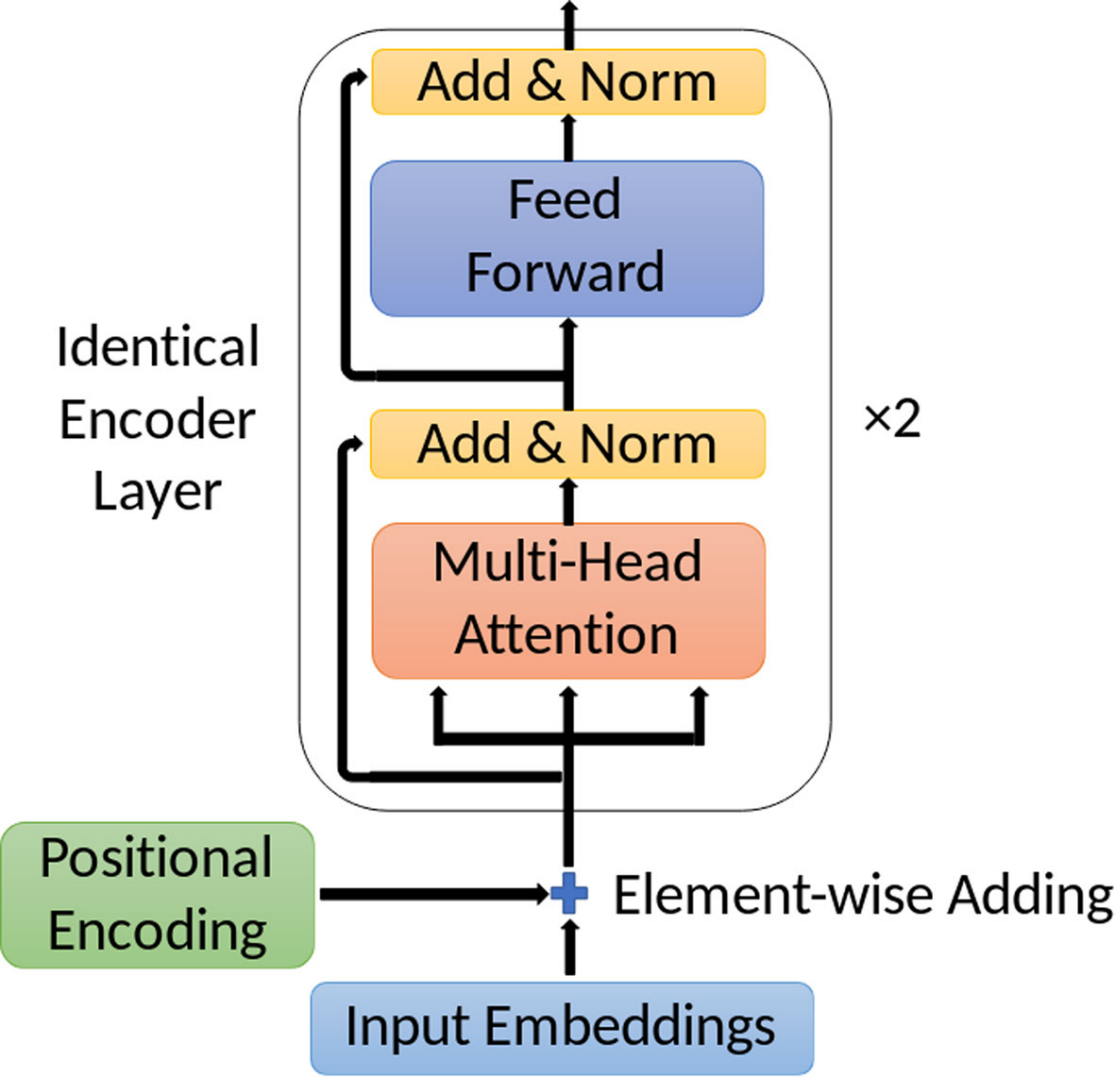
The structure of an identical encoder layer

Our attention architecture is actually the encoder of the Transformer in [18]. We only use the encoder of the Transformer since our task is to provide the correct label on the sense of the ambiguous word based on the inputs, whose complexity is not high enough for a decoder. The complete encoder layer is a stack of two identical encoder layers. The structure of one encoder layer is shown in Fig. 3.

Similar to the BiLSTM model, the input to the attention architecture is pre-trained word embeddings **X**=(**x**_1_,⋯,**x**_*T*_). Then, the attention architecture shall perform on the input to obtain an output **Y**=(**y**_1_,⋯,**y**_*T*_). In our model, both the input embeddings and the output embeddings have the same dimensions $d_{\mathbf {x}_{t}}=d_{\mathbf {y}_{t}}=d_{{model}}=200\phantom {\dot {i}\!}$.

Each layer of the encoder consists of two sub-layers: A multi-head self-attention sub-layer, and a position-wise fully connected feed-forward sub-layer. Also as shown in Fig. 3, a residual connection is applied to each sub-layer before a normalization. That is, suppose the input to one sub-layer is **X** and the functional implementation of this sub-layer is Sublayer(**X**). Then, the output of this sub-layer is LayerNorm(**X**+Sublayer(**X**)).

**Fig. 4:**
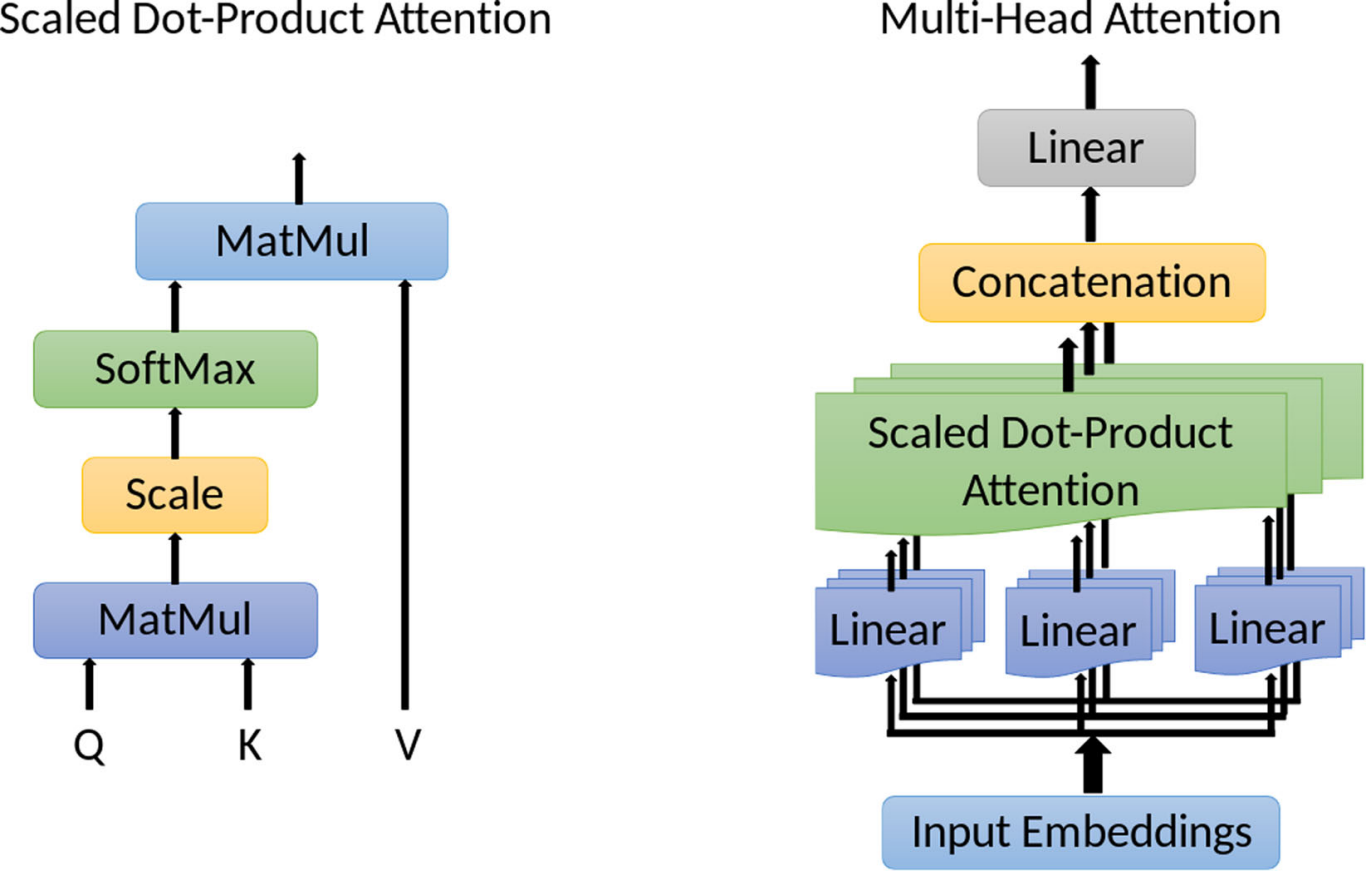
The scaled dot-product attention and multi-head self-attention

Within the multi-head self-attention sub-layer, the major attention operations are implemented by the mechanism called Scaled Dot-Product Attention, which is a self-attention mechanism. These structures are shown in Fig. 4.

We introduce the scaled dot-product attention in the next part, and subsequently the multi-head attention.

#### Scaled Dot-Product Attention

The initial inputs to the scaled dot-product attention are the input embeddings **X**=(**x**_1_,⋯,**x**_*T*_). But **X** is not the direct input: Consider **X**=(**x**_1_,⋯,**x**_*T*_) as the stack of each input embedding **x**_*t*_, which makes **X** a *T*×*d*_*model*_ matrix. Then, three matrices are generated based on **X** as the direct inputs to scaled dot-product attention as:

∙ The Query **Q**=**XW**^*Q*^, where **W**^*Q*^ is a *d*_*model*_×*d*_*q*_ matrix and hence **Q** is a *T*×*d*_*q*_ matrix.

∙ The Key **K**=**XW**^*K*^, where **W**^*K*^ is a *d*_*model*_×*d*_*k*_ matrix and hence **K** is a *T*×*d*_*k*_ matrix. And the scaled dot-product attention follows *d*_*q*_=*d*_*k*_.

∙ The Value **V**=**XW**^*V*^, where **W**^*V*^ is a *d*_*model*_×*d*_*v*_ matrix and hence **V** is a *T*×*d*_*v*_ matrix.

We will introduce the operation of the scaled dot-product attention based on each column of the matrices **Q**, **K** and **V**. As such, the intuitive meaning of each matrix can be reflected. Take the first word embedding **x**_1_ as an example: We create its query vector **q**_1_=**x**_1_**W**^*Q*^, its key vector **k**_1_=**x**_1_**W**^*K*^ and its value vector **v**_1_=**x**_1_**W**^*V*^. Then, its query vector **q**_1_ will do inner product: *p*_*t*_=**q**_1_·**k**_*t*_^*T*^ with all the key vectors **k**_1_,⋯,**k**_*T*_ generated via **k**_*t*_=**x**_*t*_**W**^*K*^ (Here, the capital T on *k*_*t*_^*T*^ means vector transformation, which has nothing to do with time steps of the input).

The scaled product *p*_*t*_ represents the amount of attention the word *w*_1_ shall put onto the word *w*_*t*_. So, “query” means a “consultation” from a word, and “key” means the own property of a word. The inner product between the query vector of one word and the key vector of another represents how important the latter word is to the former one, according to the level of the match between the key and the query.

Then, a softmax is implemented on *p*_1_ through *p*_*T*_ after dividing them by $\sqrt {d_{k}}$:

$$\begin{array}{*{20}l}(s_{1},\cdots,s_{T})&=\text{Softmax}\left(\frac{p_{1}}{\sqrt{d_{k}}},\cdots,\frac{p_{T}}{\sqrt{d_{k}}}\right)\\ &=\text{Softmax}\left(\frac{ {\mathbf{q}_{1}}^{T}\cdot\mathbf{k}_{1} }{\sqrt{d_{k}}},\cdots,\frac{{\mathbf{q}_{1}}^{T}\cdot\mathbf{k}_{T}}{\sqrt{d_{k}}}\right) \end{array} $$

Finally, a weighted summation is implemented between (*s*_1_,⋯,*s*_*T*_) and (**v**_1_,⋯,**v**_*T*_) to get the output **z**_1_ with respect to the input embedding **x**_1_ as: $\mathbf {z}_{1}=\sum _{t=1}^{T}s_{t}\mathbf {v}_{t}$.

The vectors **z**_2_,⋯,**z**_*T*_ are generated similarly with respect to **x**_2_,⋯,**x**_*T*_. Then, the stack of vectors **Z**=(**z**_1_,⋯,**z**_*T*_) is the output matrix of the scaled dot-product attention. Since vector **z** has dimension *d*_*v*_ as vector **v**, we have that the matrix **Z** is *T*×*d*_*v*_.

In addition, the inner products between vectors **q** and **k** can be represented by matrix multiplication. Combining the softmax weighted summation, we can use the following concise formula to represent the operation of scaled dot-product attention:

$${}\begin{aligned} \text{Attention}(\mathbf{X})&=\text{Attention}\left(\mathbf{X}\mathbf{W}^{Q},\mathbf{X}\mathbf{W}^{K},\mathbf{X}\mathbf{W}^{V}\right)\,=\,\text{Attention}(\mathbf{Q},\mathbf{K},\mathbf{V})\\ &=\text{Softmax}\left(\frac{\mathbf{Q}\mathbf{K}^{T}}{\sqrt{d_{k}}}\right)\mathbf{V}=\mathbf{Z} \end{aligned} $$

The reason to divide the dot product between query vectors and key vectors by $\sqrt {d_{k}}$ is that, the dot product grows large in magnitude if *d*_*k*_ is large. And a large magnitude of the dot product shall push the softmax function into a region with small gradient. As a result, the scaled dot-product attention shall divide the dot product between **q** and **k** by $\sqrt {d_{k}}$ to control the magnitude.

However, a single scaled dot-product attention will not be the final output of the attention mechanism in one identical encoder layer. Instead, the final output is based on a concatenation of multiple scaled dot-product attention mechanisms, which also generate the output tensor with the same shape as the input embeddings **X**.

#### Multi-head attention

Instead of implementing a single scaled dot-product attention, the authors of [18] implemented multiple of them in parallel. That is, multiple sets of queries, keys and values are generated based on the same input embeddings **X**, and then a scaled dot-product attention is implemented on each set in parallel. After that, the outputs from these scaled dot-product attentions are concatenated and projected linearly to get a final output. This process is shown in the right hand side of Fig. 4.

That is,

$${}\begin{aligned} \mathrm{Multi\!\,-\,\!Head \ Attention}(\mathbf{X})&=\text{Concat}(\mathbf{head}_{1},\cdots,\mathbf{head}_{h})\mathbf{W}^{O}=\mathbf{Z},\\ \text{with} \ \ \mathbf{head}_{i}=\text{Attention}_{i}(\mathbf{X})&=\text{Attention}\left(\mathbf{X}\mathbf{W}_{i}^{Q},\mathbf{X}\mathbf{W}_{i}^{K},\mathbf{X}\mathbf{W}_{i}^{V}\right). \end{aligned} $$

where the projection matrices $\mathbf {W}_{i}^{Q}\in \mathbb {R}^{d_{model}\times d_{q}}$, $\mathbf {W}_{i}^{K}\in \mathbb {R}^{d_{model}\times d_{k}}$ and $\mathbf {W}_{i}^{V}\in \mathbb {R}^{d_{model}\times d_{v}}$ for *i*=1,2,⋯,*h* are independent in different heads. The final projection matrix is $\mathbf {W}^{O}\in \mathbb {R}^{h d_{v}\times d_{model}}$. Similar to the single scaled dot-product attention, we always have *d*_*q*_=*d*_*k*_.

In this work, we apply multiple *h* values. We always set *d*_*k*_=*d*_*v*_=*d*_*model*_/*h* in the multi-head attention sub-layer. No matter what values *d*_*k*_, *d*_*v*_ are, the final output **Z** of the multi-head attention sub-layer is always a *T*×*d*_*model*_ matrix. This is because the concatenation of the heads generates a *T*×*hd*_*v*_ matrix, which is used to multiply $\mathbf {W}^{O}\in \mathbb {R}^{h d_{v}\times d_{model}}$ to produce the output $\mathbf {Z}\in \mathbb {R}^{T\times d_{model}}$. As a result, **Z** shall have the same shape as the input embeddings **X**, so that the residual connection can be implemented.

The multi-head attention is more beneficial than a single scaled dot-product attention for several reasons. By applying multiple attention heads, the final linear projection **W**^*O*^ can provide the result based on independent attention outputs, which will reduce the error rate. We will discuss this further in the “Discussion” section.

However, the final output from the multi-head attention sub-layer is not directly used as the encoder output. A feed-forward neural network operates on the output from the multi-head attention sub-layer in order to get a further filtered and projected result. We shall introduce the structure of the feed-forward network in part four of this subsection, and provide a brief analysis on why we need a feed-forward neural network on top of the attention mechanism in the “Discussion” section.

#### Position-wise feed-forward networks

Above the attention sub-layer in one identical encoder, there is the position-wise feed-forward network, whose input is the output **Z** of the multi-head attention sub-layer. The position-wise feed-forward network shall operate on each column of **Z** separately and identically. This operation consists of two linear transformations and a ReLU activation in between. That is, suppose **Z**=(**z**_1_,⋯,**z**_*T*_) with $\mathbf {z}_{t}\in \mathbb {R}^{d_{model}}$. Then, we have that

$$\begin{array}{*{20}l} \text{FFN}(\mathbf{Z})&=(\text{FFN}(\mathbf{z}_{1}),\cdots,\text{FFN}(\mathbf{z}_{T})),\\ &\text{with} \ \ \text{FFN}(\mathbf{z}_{t})=\max(0,\mathbf{z}_{t}\mathbf{W}_{1}+\mathbf{b}_{1})\mathbf{W}_{2}+\mathbf{b}_{2}, \end{array} $$

where the maximum (ReLU activation) is performed identically on each dimension.

While the linear transformations are the same for all **z**_*t*_, the parameters {**W**_1_,**W**_2_,**b**_1_,**b**_2_} can be different from layer to layer. We always have $\mathbf {W}\in \mathbb {R}^{d_{model}\times d_{model}}$ and $\mathbf {b}\in \mathbb {R}^{d_{model}}$, so that the final output FFN(**Z**) shall have the same dimensionality as the input embeddings **X**.

Then, as we mentioned at the beginning of this subsection, the same upper layers as in the BiLSTM neural network model are applied on top of the attention architecture here. That is, the output FFN(**Z**) from each of the two identical encoder layers shall play the same role as the output from each BiLSTM layer, so that structure (i) through structure (iv) may be performed right after.

Note that the attention architecture introduced in the above four parts contains no convolutional or recurrent structure to deal with sequential information. As a result, an additional method is required to make use of the order of sequence of the input words. This method is called positional encoding, which is discussed in the final part of this subsection as follows.

#### Positional Encoding

In language modeling, the order of the input sequence usually contains important information. So a language modeling system should have an efficient mechanism to make use of the order of the input sequence. Although the attention architecture in [18] has no convolutional or recurrent structure to process the sequential information, an encoding method is applied to directly encode the order of input words into the input embeddings.

That is, for the input embeddings **X**=(**x**_1_,⋯,**x**_*T*_), the positional embeddings **PE**=(**pe**_1_,⋯,**pe**_*T*_) are generated with

$$\begin{array}{*{20}l} \mathbf{pe}_{t,2i} &= \sin\left(\frac{t}{10000^{2i/d_{model}}}\right),\\ \mathbf{pe}_{t,2i+1} &= \cos\left(\frac{t}{10000^{2i/d_{model}}}\right). \end{array} $$

where **pe**_*t*,2*i*_ means the *i*’th dimension of the positional embedding **pe**_*t*_. Then a simple matrix addition **X**+**PE** provides the actual input embeddings to the attention architecture. In this way, the positional information is injected into the embedding by the sinusoid function.

The reason to choose sinusoid functions is based on the assumption that they would allow the model to easily learn to attend by relative positions, since for any fixed *k*, **pe**_*t*+*k*_ can be represented as a linear function of **pe**_*t*_.

## Results

In this section, we will introduce our experimental settings and results. We will also compare our results with the results from other papers. Both our BiLSTM model and our self-attention model produce promising performance. But in general, our BiLSTM model works better, providing the state-of-the-art performance on the MSH WSD dataset.

We used the same experimental settings for both of our approaches: The word embeddings are pre-trained with the skip-gram model by Mikolov et al. [12] on the joint dataset Wikipedia + PubMed + PMC. And all our models were trained on the MSH WSD dataset, consisting of 203 separate files. Each file contains around 200 paragraphs, which are the training corpus of a specific biomedical ambiguous word, whose location is marked out in each paragraph. However, 17 out of 203 ambiguous words do not have corresponding pre-trained embedding. As as result, our training and evaluating were performed on the remaining 186 words.

We used the same training method for these two approaches: We trained a single BiLSTM neural network model on all the 186 words simultaneously to get a **universal** WSD network. In other words, we merged all the 186 datasets into a large one and then trained the models with it. We trained one BiLSTM neural network model on each dataset of an ambiguous word *w* to get 186 **word-specific** WSD networks in total. Similarly, we trained a universal WSD attention model on the merged dataset, and 186 word-specific WSD attention models on the dataset for each ambiguous word *w*.

We found that the model using the whole paragraph as the input yields better results than that using 25 words before and after the target ambiguous word in the paragraph. However, training on the whole-paragraph input is much more expensive than that on the 25-25-word input for a deep neural network. As a result, we trained most of our BiLSTM neural network models using the 25-25-word input. That is, we chose the neural network model with best performance on the 25-25-word input, then we trained it using the input consisting of whole paragraphs. On the other hand, the training of the attention model is much more efficient than that of the deep networks. As a result, we always used the entire paragraph as the input for the attention model.

When training both universal and word-specific models with both deep network and self-attention architectures, we always randomly picked 70% of the paragraphs as the training set, while 10% and 20% of the paragraphs were used as the validation and testing sets, respectively.

Based on the above settings and the early stopping technique, our best performance reached test accuracy of 96.00%, which came from the word-specific BiLSTM neural network model with the time-step concatenation (structure (iii)) and without layer $\mathcal {C}$, trained on the whole-paragraph inputs. Then, since structure (iii) provides the best result for the BiLSTM neural network model among all the four structures, we only trained our attention model (both word-specific and universal) with structure (iii). After applying several different values of *h* in the multi-head attention, we found that the attention model in general is slightly less accurate than the neural network models, although the training is 3 to 4 times faster than that of the neural network models. Our best test accuracy of the word-specific attention model is 93.94%, coming from the model with *h*=2 trained on the whole-paragraph inputs. We show the results from our word-specific models in Table 1.

**Table 1:** Average test accuracy (in percent) by word-specific models with structure (i) through (iv), with and without layer $\mathcal {C}$

Models	with $\mathcal {C}$	without $\mathcal {C}$
Basic NN	93.40	94.25
Sum NN	93.60	94.48
Cct-V NN	94.41	94.78
Cct-T NN	94.50	94.87
Cct-T NN whole-paragh	NA	**96.00**
Attention cct-T whole-paragh	93.80	93.94

Here, we use basic NN, sum NN, cct-T NN and cct-V NN to refer to the BiLSTM neural network model with structure (i), (ii), (iii), and (iv), respectively. All of these four models were trained with the 25-25-word input. The Cct-T NN whole-paragh refers to the BiLSTM neural network model with structure (iii) trained on the whole-paragraph input. Attention cct-T whole-paragh refers to the word-specific attention model with structure (iii) trained on the whole-paragraph inputs. From Table 1, we can see that the optional layer $\mathcal {C}$ actually reduces the accuracy of word-specific models, which will be further discussed in the “Discussion” section.

Finally, we randomly choose approximately 90 percent of the paragraphs from each file as our training set, and the remaining 10 percent paragraphs are used as our testing set. Then, our best performing model, the BiLSTM neural network model under structure (iii) without layer $\mathcal {C}$, is trained with the whole-paragraph inputs. The training always stops after 50 epochs, since there is no validation set for early stopping technique. With these settings, our testing accuracy reaches 97.14%. To the best of our knowledge, this result surpasses the existing state-of-the-art WSD performance on the MSH WSD dataset [15].

Since the training of universal network is time consuming, we only train the universal BiLSTM neural network model with structure (iii), the best performing structure. The optional layer $\mathcal {C}$ significantly improved the performance of our universal neural network model: After applying the optional layer $\mathcal {C}$, the test accuracy of our universal neural network model increases from 80.72% to 88.75%. We believe that the target ambiguous word is re-emphasized by the optional layer $\mathcal {C}$, which significantly improves the prediction accuracy of the universal network. This issue will be discussed further in the “Discussion” section.

We also trained our universal attention models with structure (iii) on several different values of *h* in the multi-head attention. The one with *h*=4 provides the best test accuracy of 82.40% with layer $\mathcal {C}$, and 63.50% without it. It is interesting that the best universal attention model has four heads in the multi-head attention architecture, while the best word-specific one only has two. We will provide our analysis on why the universal attention model needs more heads than the word-specific ones in the “Discussion” section.

Among the 203 biomedical ambiguous words in the MSH WSD dataset, 20 of them have often been used as comparison baselines for WSD models. For instance, Festag et al. showed their disambiguation result on these 20 words in [14], applying a Recurrent Convolutional Neural Network (RCNN) as their WSD model. Jimeno-Yepes et al. provided multiple approaches to biomedical WSD in [25] with these 20 words. Their best performance came from a supervised Naive Bayes (NB) model with WEKA data mining package. We will compare the results from both our BiLSTM model and our attention model with the result from the RCNN model in [14], and the result from the Supervised NB model in [25], which are shown in Tables 2 and 3.

**Table 2:** Test accuracies (in percent) on 20 words from our word-specific models and the two baselines

Words	Basic NN	Sum NN	Cct-V NN	Cct-T NN	Cct-T Atten	Cct-T NN wp	Baseline 1	Baseline 2
AA	100.00	100.00	100.00	100.00	97.20	100.00	96.00	98.99
Astragalus	100.00	100.00	100.00	100.00	100.00	100.00	100.00	97.47
CDR	100.00	100.00	100.00	100.00	96.40	100.00	97.00	100.00
Cilia	96.15	92.31	96.15	96.15	93.80	92.00	82.00	94.87
CNS	91.18	94.12	91.18	97.06	97.20	100.00	98.00	98.48
CP	96.30	96.30	96.30	96.30	100.00	100.00	97.00	98.32
dC	97.22	100.00	97.22	97.22	100.00	100.00	98.00	98.48
EMS	97.14	97.14	97.14	97.14	100.00	100.00	98.00	100.00
ERUPTION	97.14	100.00	94.29	100.00	100.00	100.00	100.00	100.00
FAS	100.00	100.00	100.00	100.00	100.00	100.00	100.00	99.49
Ganglion	91.67	91.67	94.44	88.89	88.90	91.18	90.00	93.43
HCl	97.22	97.22	97.22	97.22	100.00	100.00	100.00	100.00
INDO	100.00	100.00	100.00	100.00	100.00	100.00	87.00	99.18
lymphogranulomatosis	94.44	100.00	94.44	100.00	95.80	100.00	83.00	83.33
MCC	87.50	100.00	100.00	100.00	100.00	100.00	97.00	100.00
PAC	100.00	100.00	100.00	90.91	100.00	100.00	94.00	100.00
Phosphorus	86.11	86.11	83.33	83.33	91.70	94.44	78.00	83.84
Phosphorylase	86.67	90.00	86.67	90.00	78.10	90.00	52.00	87.35
TMP	100.00	100.00	100.00	100.00	100.00	100.00	81.00	98.00
TNT	100.00	100.00	100.00	100.00	97.20	100.00	98.00	99.49
Average	95.94	97.24	96.42	96.71	96.82	**98.38**	91.30	96.54

Baseline 1 is the RCNN model by Festag in [14], and baseline 2 is the Naive Bayes Model by Jimeno-Yepes et al. in [25]. Cct-T Atten means the word-specific attention model with structure (iii), and Cct-T NN wp means the word-specific BiLSTM neural network model with structure (iii) trained on whole-paragraph inputs. All the word specific models here (deep network and attention) are equipped with layer $\mathcal {C}$

**Table 3:** Test accuracies (in percent) on 20 words from our universal models and the two baselines

Words	Univ NN	Univ Attention	Baseline 1	Baseline 2
AA	100.00	100.00	96.00	98.99
Astragalus	100.00	100.00	100.00	97.47
CDR	85.19	82.10	97.00	100.00
Cilia	80.77	79.80	82.00	94.87
CNS	97.06	86.10	98.00	98.48
CP	98.15	100.00	97.00	98.32
dC	94.44	82.40	98.00	98.48
EMS	91.43	98.80	98.00	100.00
ERUPTION	97.14	91.10	100.00	100.00
FAS	100.00	88.10	100.00	99.49
Ganglion	86.11	73.30	90.00	93.43
HCl	91.67	94.10	100.00	100.00
INDO	100.00	100.00	87.00	99.18
lymphogranulomatosis	88.89	75.80	83.00	83.33
MCC	83.33	92.80	97.00	100.00
PAC	81.82	81.10	94.00	100.00
Phosphorus	83.33	75.70	78.00	83.84
Phosphorylase	70.00	66.40	52.00	87.35
TMP	100.00	78.30	81.00	98.00
TNT	94.44	78.20	98.00	99.49
Average	91.19	86.21	91.30	96.54

The Univ NN means the universal BiLSTM neural network model and the Univ Attention means the universal attention model. All our universal models (deep network and attention) are trained without layer $\mathcal {C}$

Table 2 shows the results from our word-specific models (both BiLSTM neural networks and attention) and the two baselines. According to the experience described above, all our word-specific models were equipped with layer $\mathcal {C}$. Our word-specific attention model has *h*=2 in the multi-head attention. We can see that our word-specific BiLSTM neural network models with all four structures outperform the two baselines, and achieve the accuracy higher than 95%. Our word-specific attention model performs almost as good as Baseline 2, and even better than Baseline 1. In comparison to the baselines, our word-specific BiLSTM neural network model with structure (iii) trained on whole-paragraph inputs achieves higher accuracy on some difficult words such as *Phosphorylase*, which have very similar senses and are therefore difficult to disambiguate [14]. As we can see, for these 20 words, our word-specific deep network model with structure (ii) performs on average better than that with structure (iii) by 0.5%. Nevertheless, this does not hold true when we averaged the results on all the 186 words. Because of that, we also evaluated an ensemble of all our four structures. This gave us an interesting insight into the dataset, which will be further explained in the next section.

Table 3 shows the results from our universal models (both BiLSTM neural networks and attention) and the two baselines. Both of our two universal models are under structure (iii) with *h*=4 in the multi-head attention and trained without layer $\mathcal {C}$. We can see that our universal deep network model performs almost as good as the model from Baseline 1. However, we found that our universal attention model failed to provide results with satisfying accuracy. It seems that the attention model is only suitable for word-specific tasks. We believe that this is because the attention structure is too simple to process the large, merged data containing multiple ambiguous words. We shall discuss this issue in detail in the next section.

## Discussion

In this section, we provide further analysis and comparison on WSD methods based on the BiLSTM neural network model and the self-attention model. Then, we will discuss some issues of the dataset. We believe that some labels may not be accurate, which places an upper bound on the accuracy.

### Why does LSTM cell work?

In this subsection, we provide mathematical analysis on how the gate operations in an LSTM cell resolve gradient vanishing (or blow-up) along the time steps.

The purpose of gate operations in an LSTM cell is to ensure that the memory is not impacted by gradient vanishing (or blow-up), which is the major defect of a typical RNN. Mathematical analysis shows that the gradient *∂***o**_*t*+*K*_/*∂***c**_*t*_ should not vanish (or blow-up) when *K* is large. Using the chain rule of partial derivative into Eqs. (1), (2), (3), (4) and (5) in the Methods” section, we have

$$\begin{array}{*{20}l} \frac{\partial \mathbf{o}_{t+K}}{\partial \mathbf{c}_{t}}=\frac{\partial \mathbf{o}_{t+K}}{\partial \mathbf{c}_{t+K}}\cdot\prod_{k=1}^{K}\frac{\partial \mathbf{c}_{t+k}}{\partial \mathbf{c}_{t+k-1}}=W_{co}\cdot\prod_{k=1}^{K}\mathbf{f}_{t+k}. \end{array} $$

It is easy to see that setting **f**_*t*_≡1 is a simple way to avoid gradient vanishing. According to Eq. (2), the direct dependent variables of **f**_*t*+*k*_ are **x**_*t*+*k*_ and **h**_*t*+*k*−1_. Due to the variance from the inputs and the complicated interactions among the gates in LSTM cells, vectors **x**_*t*+*k*_ and **h**_*t*+*k*−1_ possess time-step independent distributions. Accordingly, **f**_*t*+*k*_ can be inferred as time-step independent as well. Therefore, when *K* is large, the number of **f**_*t*+*k*_ with extremely large absolute value will be almost equal to the number of those with extremely small absolute value due to time-step independence, and hence they shall cancel each other in the product $\prod _{k=1}^{K}\mathbf {f}_{t+k}$. As a result, *∂***o**_*t*+*K*_/*∂***c**_*t*_ will not vanish or blow-up when *K* is large. This performance guarantees that **c**_*t*_ can be efficiently updated by cross-entropy error in many steps ahead, which explains why the LSTM cell can remember long-term dependencies.

### Why does time-step concatenation work best?

In our experiments, we hope the contextual information can be captured by our BiLSTM network in an explicit process. In order to do so, we adapted the ideas behind the ELMo model in [16], combining with our own upper layer design to fit the WSD task better. As shown in the “Methods” and “Results” sections, the time-step concatenation structure performs the best.

Our explanation is, concatenation of BiLSTM outputs along the time-step would better preserve both output layers, comparing to the basic BiLSTM model and the weighted summation one. As a result, the max-pooling operation will have a better chance to capture the information from the first layer output, and hence increasing the utilization of the first layer nodes of the BiLSTM. In contrast, although the output vectors from both layers are preserved as well by the concatenation along the vector, this concatenation increases the vector’s dimension and the max-pooling dimension. Due to this reason, redundant information may be included in the max-pooled vector, leading to less useful information being out-weighted. The experimental results coincide with this analysis: Although both of the concatenation models perform better than the basic BiLSTM model and the weight summation one, the time-step concatenation structure outperforms the vector concatenation one a little bit.

### Why do we desire an attention model?

As mentioned in the “Results” section, our self-attention model performs three to four times faster than our neural network model. Yet, the computational efficiency does not constitute the entire consideration on the attention models. According to the initial work [18] of the self-attention model, three aspects are considered when evaluating a language processing system: the computational efficiency, the number of sequential operations required, and the path length that signals have to travel in the system. But since the LSTM neural network already possesses excellent path length, we do not need a self-attention model just in seeking of a longer path length. So, we only discuss the first two aspects here.

When comparing the computational complexity between different structures, people always use the operations needed in each layer, i.e., the computational complexity per layer, as the measurement. According to the attention structure introduced in the “Methods” section, we can see that the major operations in an identical encoder layer come from the matrix multiplications. So by a routine calculation, we can get the computational complexity per attention layer as $\mathcal {O}(T^{2}\cdot d_{model})$, where *T* is the length of the input and *d*_*model*_ is the dimension of the embedding. On the other hand, the computational complexity per recurrent layer is $\mathcal {O}(T\cdot {d_{model}}^{2})$; and the computational complexity per convolutional layer is $\mathcal {O}(k\cdot T\cdot {d_{model}}^{2})$, where *k* is the kernel size of convolutions. For most of the state-of-the-art language models, the embedding size *d*_*model*_ is around 150 to 500, which is necessarily larger than the average temporal length *T* of the inputs. As a result, we shall have *T*^2^·*d*_*model*_≪*T*·*d*_*model*_^2^≪*k*·*T*·*d*_*model*_^2^. This relationship explains why the attention model operates much faster, and why the convolutional neural network operates much slower than the recurrent neural network.

By the number of sequential operations required, it means the amount of operations needed on each time step per layer. In this case, the recurrent operation has to be implemented at each time step, rendering an RNN with $\mathcal {O}(T)$ sequential operations. The convolutional operation with kernel width *k* and stride *k* brings $\mathcal {O}(T/k)$ sequential operations to a typical CNN language model (different from the stride-one convolutions in vision, where a CNN language model usually has a stride size same as the kernel width). In contrast, the matrix multiplication in an identical encoder layer covers all time steps, providing the self-attention model with $\mathcal {O}(1)$ sequential operations. As a result, we can see that the self-attention model possesses the advantage towards RNN or CNN structures in terms of sequential operations. Since sequential operations decide the actual computing process in CPUs, an attention model as a result necessarily reduces the true CPU time required for training.

Therefore, although being slightly less accurate than a deep recurrent neural network, we still desire an attention model to perform word sense disambiguation.

### Why does an attention model fail to yield better accuracy than deep networks?

Although being efficient on sequential inputs, a self-attention model is limited in some aspects. According to the experimental results, the major defect of an attention model is the unsatisfactory accuracy compared to deep neural network models.

We need to pay attention to two facts: First, in the case of word-specific WSD models, our best attention model yields test accuracy that is 2 percent lower than that of our best BiLSTM neural network model. In the case of universal models, our attention model failed to provide a satisfactory result. Second, the best universal attention model has four heads in the multi-head attention architecture, while the word-specific attention model only has two. We believe that these facts from our experiments provide a plausible reason of the sub-optimal accuracy of an attention model: The attention structure is too simple to learn complicated representations from big and complex data.

As the analysis we did in the above subsection, the complex structure of each LSTM cell enables the network to learn complicated temporal representations from the input data. The combination of various activation functions within each cell as well as the stack of layers enables the deep network to establish complicated decision regions in the input space. In contrast, the attention model does not involve an activation function. The major operations of a multi-head attention are paralleled matrix multiplications. As a result, we believe that the representation from an attention model is quite restricted by the simplicity of its structure. This restricted ability on representation learning makes an attention model reluctantly provide satisfying results on small, word-specific datasets, yet failed to perform on the merged, complicated big dataset for universal learning, even with an increased number of heads in the multi-head attention architecture.

We can clearly see the trade-off made by the attention model: sacrificing accuracy in order to obtain computational efficiency. As a result, comparing to the efficiency, it is an acceptable defect for the attention model to perform less accurate than the deep network.

### Why do we need a feed-forward network on top of the attention structure?

Note that, the only activation function in an identical encoder layer is the ReLU function in the feed-forward sub-layer on top of the self-attention structure. Similar to a convolutional neural network or a recurrent neural network, the activation functions serve as filters, which eliminate the redundant information and keep the relative representations. As a result, the errors and redundant information from the self-attention sub-layers shall be filtered out by the ReLU gate in the feed-forward sub-layer, making the final output more efficient for disambiguating. We believe that this is the major reason for the self-attention model to have a feed-forward network on top of the attention sub-layer.

It is often desirable to keep a balance between performing accuracy and computational efficiency. As a result, the feed-forward network in [18] consists of only two matrix transformations, in which the first transformation is equipped with the ReLU function. As such, the activation functions in the feed-forward network serve as filters to improve the performance, while avoiding increasing the computational complexity of the self-attention model too much. Otherwise, a deep feed-forward sub-layer would consume the computational efficiency obtained by the attention structure.

### Why is multi-head attention better than a single scaled dot-product attention?

We believe that, the reason for researchers in [18] to concatenate the output from each single scaled dot-product attention to build a multi-head structure is that, the multi-head attention enables a “vote”, or averaging on the output from each single attention structure, so that the chance of making mistakes is reduced.

In addition, the parallel performance of each single attention structure shall enable the attention model to build flexible decision regions and learn complicate representations, which to a certain extent mediates the low ability of the attention model on representation learning. Meanwhile, the parallel performance in the multi-head structure avoids increasing the computational complexity: matrix multiplications can be performed in parallel in multiple units in a GPU. Thus, a machine equipped with multiple GPUs can speed up the attention model even more.

The above analysis comprehensively compared our deep BiLSTM neural network model and our self-attention model on word sense disambiguation tasks. In general, a deep neural network model provides more accurate results especially on big datasets, while a self-attention model performs much faster and can get satisfactory results, especially on small datasets. Researchers in the future may take our analysis into consideration when choosing a WSD architecture for a specific data set.

### Is an universal WSD system possible?

As shown in the “Results” section, the optional layer $\mathcal {C}$ significantly improves the performance of our universal WSD model. However, when we add the optional layer $\mathcal {C}$ back to our word-specific models, it is surprising that the prediction accuracies are slightly reduced.

We believe that when training a universal network, the optional layer $\mathcal {C}$ will put further emphasis on the target ambiguous word to the dense layer, thereby leading to a more meaningful representation in the dense layer on the contextual information. In contrast, when training on the dataset for a single ambiguous word, further emphasis is redundant and may actually overwhelm some useful information in the max-pooled vector **h**, therefore slightly disturbing the dense layer on generating contextual information representations.

As far as we know, our universal WSD network is the first universal model in biomedical word sense disambiguation. Our results indicate that a universal model is feasible for NLP tasks. We hope that our research can draw research community’s attention to the universal word sense disambiguation system in the future.

### Is there any mis-labeling in the MSH WSD dataset?

After looking into the prediction output from neural networks with all the four upper layers, we found an interesting phenomenon: When testing with some specific paragraphs, the less accurate upper layer structures (i, ii, iv) on average even outperformed the best one (iii).

To further examine this phenomenon, we created two ensemble methods on neural network models: The first ensemble method makes neural networks with structure (i) through (iv) do a majority voting on the output. The second method implements a weighted voting, where the network with better performance on a validation set has a larger weight. Ensemble methods resulted in a small gain in accuracy (up to 0.4%).

Then, we further looked into the mistakes made by the networks. It appears that all our models agree on the same wrong label in 190 cases (3% of the test set). Examination of these words and their corresponding paragraphs shows that the disambiguation in these cases are very challenging even to human experts. For example, the word *Medullary* has two concepts in the UMLS:

- *Adrenal Medulla*, inner part of the adrenal gland, (Body Part, Organ, or Organ Component)
- *Medulla Oblongata*, also part of the brain, (Body Part, Organ, or Organ Component).

Both of these concepts relate to parts of the brain and have a similar meaning, rendering disambiguation challenging.

Other words can be argued to have more than one correct sense in the given paragraph. The word *Laryngeal* has two corresponding concepts in the UMLS:

- *Larynx*, irregularly shaped, musculocartilaginous tubular structure, (Body Part, Organ, or Organ Component)
- *Laryngeal Prosthesis*, a device, activated electronically or by expired pulmonary air, which simulates laryngeal activity and enables a laryngectomized person to speak, (Medical Device).

In following paragraph from the dataset of *Laryngeal*:

*“A comparative study of speech after total laryngectomy and total laryngopharyngectomy. Quality of voice is an important factor in the consideration of treatment for advanced ****laryngeal**** cancer. This prospective study compared the speaking proficiency of patients who used the Blom-Singer valve after total laryngectomy and after total laryngopharyngectomy with jejunal graft reconstruction with that of a group of normal subjects. The total laryngectomy group demonstrated excellent communication ability both face-to-face and on the telephone. They exhibited superior scores for objective intelligibility, subjective intelligibility, acceptability, and intonation when compared with the total laryngopharyngectomy group....”*

This paragraph is labeled with the second meaning (Laryngeal) in the UMLS. However, one can argue that the first meaning (Larynx) would be more appropriate, which is the meaning predicted by our networks. Those examples suggest that there may be an upper bound of accuracy on this dataset, which we think is around 97%.

## Conclusions

In this paper, we propose both word-specific and universal WSD models using a deep BiLSTM neural networks and self-attention architectures, with four different adjustments on the models’ outputs and an optional concatenation layer. Experiments showed that the contextual information is sufficient for word sense disambiguation, and can be efficiently learned by deep networks as well as attention architectures. Furthermore, obtaining an explicit and wide representation of the contextual information improves the disambiguation accuracy significantly. Finally, re-emphasizing the target ambiguous word is crucial to our universal WSD system.

In addition, we believe that good word embeddings are essential to all NLP tasks. However, being the state-of-the-art language model, the skip-gram negative sampling (SGNS) model does not capture the sense of word when training their embeddings [12]. Therefore, we hope to establish a language model using deep neural networks, so that not only the embeddings of words but also the embeddings of senses is established. As such, further semantic information can be captured naturally by both the word embeddings and the deep network.

## Data Availability

The MSH WSD dataset used in this study is publicly available at https://wsd.nlm.nih.gov/collaboration.shtml. The code of the Skip-gram model is available at http://evexdb.org/pmresources/vec-space-models/. The source code of the models is available upon request.

## Abbreviations

BiLSTM: Bidirectional long short-term memory
CNN: Convolutional neural network
CUI: Concept unique identifier
LSTM: Long short-term memory
NER: Named-entity recognition
NLP: Natural language processing
RNN: Recurrent neural network
SGNS: Skip-gram negative sampling
SVM: Support vector machine
UMLS: Unified medical language system
WSD: Word sense disambiguation
